# Optimization of tomotherapy treatment planning for patients with bilateral hip prostheses

**DOI:** 10.1186/1748-717X-9-43

**Published:** 2014-02-04

**Authors:** David Chapman, Shaun Smith, Rob Barnett, Glenn Bauman, Slav Yartsev

**Affiliations:** 1 London Regional Cancer Program, London Health Sciences Centre, 790 Commissioners Road East, London, ON N6A 4L6, Canada; 2 Departments of Oncology and Medical Biophysics, Western University, London, ON N6A4L6, Canada

**Keywords:** Tomotherapy, MVCT, Prostheses

## Abstract

**Background:**

To determine the effect of different imaging options and the most efficient imaging strategy for treatment planning of patients with hip prostheses.

**Methods:**

The planning kilovoltage CT (kVCT) and daily megavoltage CT (MVCT) studies for three prostate cancer patients with bilateral hip prostheses were used for creating hybrid kVCT/MVCT image sets. Treatment plans were created for kVCT images alone, hybrid kVCT/MVCT images, and MVCT images alone using the same dose prescription and planning parameters. The resulting dose volume histograms were compared. The orthopedic metal artifact reduction (O-MAR) reconstruction tool for kVCT images and different MVCT options were investigated with a water tank fit with double hip prostheses. Treatment plans were created for all imaging options and calculated dose was compared with the one measured by a pin-point ion chamber.

**Results:**

On average for three patients, the D_35%_ for the bladder was 8% higher in plans based on MVCT images and 7% higher in plans based on hybrid images, compared to the plans based on kVCT images alone. Likewise, the D_35%_ for the rectum was 3% higher than the kVCT based plan for both hybrid and MVCT plans. The average difference in planned D99% in the PTV compared to kVCT plans was 0.9% and 0.1% for MVCT and hybrid plans, respectively. For the water tank with hip prostheses phantom, the kVCT plan with O-MAR correction applied showed better agreement between the measured and calculated dose than the original image set, with a difference of -1.9% compared to 3.3%. The measured doses for the MVCT plans were lower than the calculated dose due to image size limitations. The best agreement was for the kVCT/MVCT hybrid plans with the difference between calculated and measured dose around 1%.

**Conclusion:**

MVCT image provides better visualization of patient anatomy and hybrid kVCT/MVCT study enables more accurate calculations using updated MVCT relative electron density calibration.

## Introduction

As the population ages, the number of patients presenting for radiotherapy with hip prostheses is expected to increase. According to the Canadian Joint Replacement Registry (CJRR), in 2006–07 there were 24,253 hip replacement surgeries performed in Canada, up 60.2% from 1996–97 [1]. The hip prostheses are typically made of high atomic number materials producing severe artifacts such as streaking and blurring in kilovoltage computed tomography (kVCT) images. These artifacts prevent accurate contour delineation and alter image density values required for accurate dose calculation.

Recent advances in imaging include newer CT reconstruction algorithms to minimize artifacts from CT imaging. In addition, cone beam and fan beam megavoltage CT images are available on treatment units. Images acquired at these energies are less subject to imaging artifacts as the primary mode of interaction at megavoltage energies is Compton (dependent on electron density) instead of photoelectric effects (dependent mainly on cube of atomic number) seen with kilovoltage energies. Magnetic resonance imaging (MRI) is often used in providing information for prostate, bladder, and rectum contours due to its better soft tissue resolution [2].

A significant amount of research has been performed on methods to reduce image artifacts in kVCT images. Wang *et al.* proposed a fast iterative algorithm that utilizes intermediate reconstruction to improve image quality [3] and Zhang *et al.* suggested pre-processing projection data for application in cone-beam CT images [4]. These tools are now routinely available for clinical use on CT-Simulators. For example, the CT-Simulator used at our institution incorporates an orthopedic metal artifact reduction (termed O-MAR) tool (Philips Healthcare, Andover, MA). O-MAR may be applied to kVCT scans when the patient has high density metal implants [5]. Recently, it has been shown that metal artifacts are significantly reduced by imaging patients with a megavoltage CT (MVCT) scanner [6-8]. Theoretically, MVCT images can also be used for dose calculation in the presence of high atomic number materials such as hip prostheses [9].

Helical tomotherapy (HT) is a relatively new approach in radiation treatment of cancer patients [10]. HT combines intensity-modulated helical radiation treatment delivery with a 6 MV beam and CT imaging with 3.5 MV x-rays to deliver precise image-guided treatment plans. MVCT is currently used to register patient positioning prior to treatment and to verify dose delivery by recalculating the dose distribution using fluence and the MVCT image set of the same day. Linear accelerators incorporating cone-beam CT capability (MVision, Siemens Medical Solution, Concord, CA) also have the potential to acquire MVCT images [11].

It is the aim of this paper to determine the most effective treatment planning strategy for patients with bilateral hip prostheses in terms of accurate dose calculation and delivery. Different planning options were analyzed using the imaging data for three patients and a specially designed phantom. The strategies considered include: using kVCT images alone, with or without a correction algorithm (O-MAR) applied; using hybrid kVCT and MVCT images, and using MVCT images alone for treatment planning.

## Materials and methods

### Effect of imaging modality on patient treatment plan

**Figure 1:**
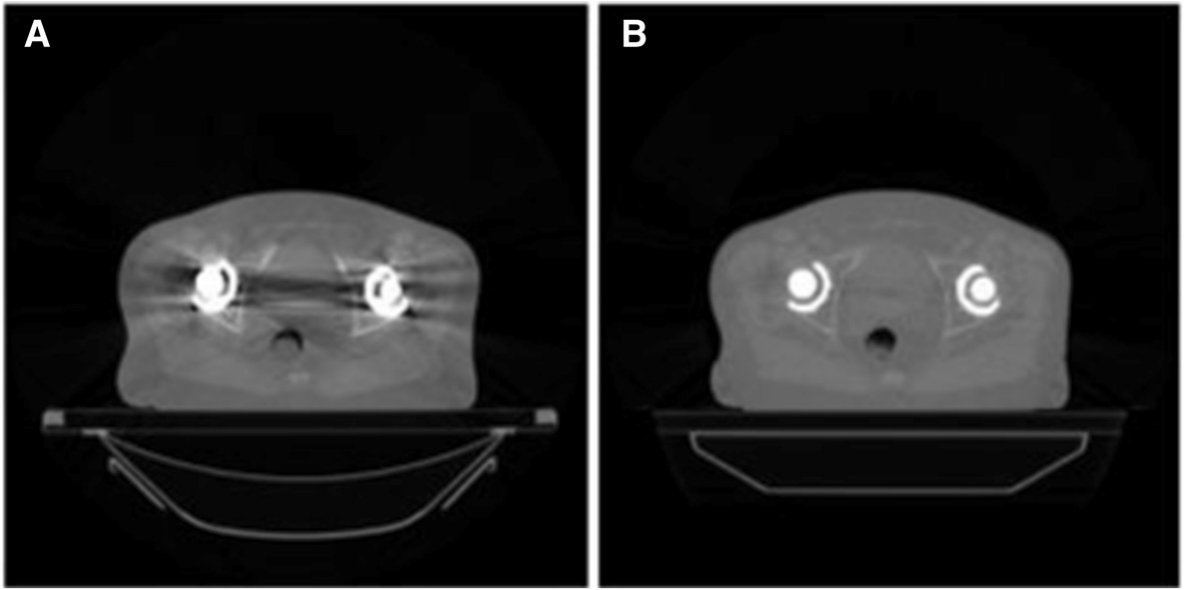
**Comparison of A) kVCT and B) hybrid kVCT/MVCT image sets.** The addition of MVCT data greatly reduces imaging artifacts.

Three prostate cancer patients with bilateral hip prostheses treated on an HT unit at the London Regional Cancer Program, London, Ontario, Canada were used for this study. The patients were immobilized using a conventional double-leg immobilization device [12] and were instructed to empty their bladder and drink 400 ml of water one hour before the treatment and try to empty their bowels. The patients initially underwent imaging on pelvis protocol (120 kVp, 50 mA) with 3 mm inter-slice spacing on a 16-slice helical kVCT simulator (Brilliance Big Bore; Philips Healthcare, Andover, MA). The presence of strong artifacts in the pelvic region due to the hip prostheses made reliable target contouring impossible (see Figure 1A). All patients were also imaged with the HT MVCT scanner using coarse (6 mm inter-slice distance) scan mode and the Planned Adaptive Software (Accuray Inc. Sunnyvale, CA) was used to merge MVCT information with kVCT image data to create hybrid image sets with 3 mm inter-slice spacing automatically interpolated from 6 mm slice MVCT data.

The HT MVCT scanner has a field of view of 40 cm, so the area outside the MVCT imaging diameter was filled with kVCT data for the purposes of dose calculation. The hybrid image sets were then transferred to Pinnacle treatment planning software (TPS) version 8.0 m (Philips Healthcare, Andover, MA) for contouring by radiation oncologists. MVCT studies were also transferred to the Pinnacle TPS and the contours drawn on the hybrid image sets were transferred to the separate kVCT and MVCT studies. This procedure provided the possibility for unambiguous organ contouring but required several steps of data transfer. The slice spacing in the kVCT and hybrid image plans was 3 and 6 mm in the MVCT image plans. The different energies of radiation used in kVCT and MVCT imaging results in different interactions of x-rays with materials therefore different electron calibration curves must be used to convert image Hounsfield unit (HU) values to electron density. The kVCT image value to density table (IVDT) was used for kVCT images and the MVCT IVDT was used for hybrid and MVCT images.

HT treatment plans were calculated using the Tomotherapy Planning Station software version 3.2 (Accuray Inc. Sunnyvale, CA) which utilizes a proprietary inverse planning optimization algorithm [13,14]. The dose distribution is calculated based on the prescription dose to the target, region of interest (ROI) constraints, and settings specific to the HT system such as modulation factor and pitch. The modulation factor (MF) is defined as the ratio between the greatest and the average (excluding closed leaves) beamlet fluence. Increasing the MF gives the software more control over optimization but requires a slower gantry rotation period, resulting in a longer treatment time. The pitch value is the ratio between couch distance traveled per gantry rotation and the field width. Increasing the pitch value reduces overlap of beams from subsequent rotations.

**Figure 2:**
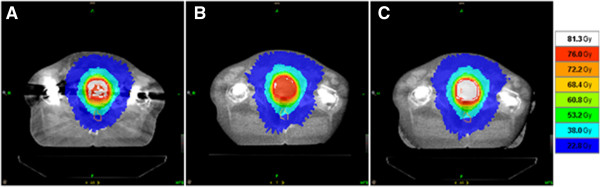
Calculated dose distribution of plans based on A) kVCT, B) MVCT and C) hybrid kVCT/MVCT image sets.

To compare the effects of employing different treatment planning images, three image sets for each patient were created: kVCT images alone, hybrid kVCT/MVCT images, and MVCT images alone. The three image sets and respective contours for each patient were transferred from the Pinnacle TPS to the Planning Station via the DICOM RT protocol. All plans were optimized using the same dose prescriptions, constraints, MF, and pitch values [15]. Treatment optimization time was also kept constant at 200 iterations. The normal dose grid option was used with calculation grid voxels of 4.6 × 4.6 × 3 mm^3^ used in kVCT and hybrid image plans. The calculation grid voxels were 3.1 × 3.1 × 6 mm^3^ in the MVCT image plans. The calculated dose distributions for each image set were investigated and a comparison of the dose distributions in the transverse plane for patient 2 is shown in Figure 2. The resulting dose volume histograms (DVH) for each treatment plan were analyzed to determine the success of avoiding critical structures such as the bladder and rectum and delivering the prescribed dose to the planning target volume (PTV). Calculated doses to the bladder and rectum at volumes of 5%, 10%, 25%, 35%, and 50% and doses to the PTV at volumes of 1% (maximum dose to PTV) and 99% (minimum dose to PTV) were analyzed for the calculated treatment plans for each patient. Unlike the kVCT images, the patient MVCT studies did not image the entire patient volume therefore there were portions of the anatomy that were not imaged. Thus we also calculated the absolute volumes that corresponded to 5%, 10%, 25%, 35%, and 50% of the bladder and rectum volume in the MVCT studies for each patient and the doses to these volumes were found in the kVCT and hybrid image plans in order to properly compare the treatment plans. The entire PTV volume was imaged in all three treatment plans therefore there was no need to calculate absolute volumes to compare dose. These data were obtained from the cumulative relative DVH for each treatment plan.

### Measurement in water phantom

To compare the effect of using different treatment planning images on treatment delivery, simulations were conducted using a water tank phantom fit with two hip prostheses (DePuy Orthopaedics Inc, Warsaw, IN). The prostheses had stems made of porous coated titanium and cups made of a reflection titanium shell, an ultra-high molecular weight polyethylene (UHWMPE) liner and a cobalt-chromium (CoCr) head. The prostheses were secured in the water tank phantom at distances and orientations that simulated patient anatomy.

**Figure 3:**
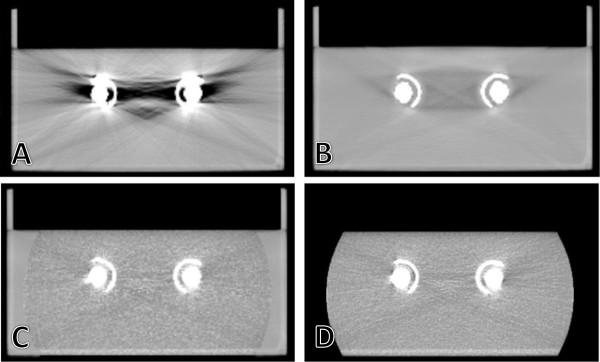
**Image sets of water tank with hip prostheses.** Comparison of **A)** original kVCT, **B)** O-MAR corrected kVCT, **C)** kVCT/MVCT hybrid, and **D)** MVCT images of the phantom with the hip prostheses inserted.

The water tank phantom was imaged both on a kVCT scanner and the HT MVCT unit. To correct for the artifacts present in the kVCT scan, the O-MAR reconstruction algorithm was applied, which greatly improved the image quality as shown in (Figure 3A,B). The DICOM sets for both the original kVCT scan and the same scan with the O-MAR correction applied were transferred to the Tomotherapy TPS as two separate “patients”. The PTV, rectum, and bladder were contoured as three cylinders. Images were acquired on the MVCT scanner for each in the coarse, normal, and fine modes (6, 4, and 2 mm inter-slice spacing, respectively). In total eleven phantom image sets were created: kVCT alone with and without O-MAR applied, MVCT alone with spacing of 2, 4, and 6 mm between slices, and hybrid kVCT/MVCT images created with each of the three MVCT image options for both the original kVCT and the kVCT with O-MAR artifact correction. The image sets were transferred to Pinnacle via the DICOM RT protocol for contouring. The contours of the PTV, bladder and rectum were then copied on to each of the hybrid and MVCT image sets and exported to the Tomotherapy planning station. Because there was no difference between the MVCT scans for the corrected or original patient, only three MVCT studies were used for planning. Pinnacle transfers contours between image sets with different inter-slice spacing by autocontouring the 3D shape enclosed by the original contours and applying the resulting contours on a specific slice. All plans were calculated using the same dose prescription and region of interest constraints. The calculation grid voxels were 3.1 × 3.1 × 6 mm^3^, 3.1 × 3.1 × 4 mm^3^, and 3.1 × 3.1 × 2 mm^3^ in the MVCT coarse, normal, and fine image plans, respectively. The altered density matrices employed by plans based on different images affect both the final dose calculation accuracy and cause the optimizer to change the planned fluence. Therefore each of the phantom plans was recalculated on the coarse hybrid image created with the OMAR correction applied using the Tomotherapy Planned Adaptive software. The difference between this verification plan and the original plan is due to the density matrices, and the deviations between verification plans is caused by planned fluence changing for each plan.

The procedure “to treat” the phantom was designed to mimic patient treatment. The phantom was placed on the HT couch and aligned with the laser positions specified for treatment. The phantom was scanned using the HT MVCT unit, the image was registered with the treatment planning study and positioning correction shifts were applied if necessary. The treatment was performed and a dose measurement was taken with a pinpoint ion chamber (Wellhöfer, Model CC01, 0.01 cm^3^, Uppsala, Sweden) at a point corresponding to the center of the PTV to compare measured and calculated doses. This process was performed for each of the 11 treatment plans.

To evaluate the influence of the presence of high density material, a kVCT image of the phantom without the hip prostheses was taken. Three MVCT scans, using the coarse, normal and fine slice widths were also taken, and a hybrid image set using each width was created. Using the same contours, dose prescription and constraints, a plan was created for each image set and dose measurements performed.

## Results

The results from the patient treatment plans obtained with the same optimization procedure for three types of image sets are shown in Table 1. Generally speaking, the doses calculated in treatment plans using MVCT images were higher than the doses calculated in the kVCT and hybrid image treatment plans. The biggest difference noted was in terms of dose to bladder. The calculated D_35_ (dose to 35% of the volume) in the bladder for patients 1 and 3 differed by -3.2% and 1.2% for MVCT and by 2.7% and 0.3% for hybrid image plans compared to the calculated dose in the kVCT image plans. Mean planned D_35_ in the rectum was within 2% of the kVCT plan dose in both the MVCT and hybrid image plans for patient 1 and the hybrid plan for patient 3, but the MVCT plan for patient 3 had a dose 8.6% lower than the kVCT plan. As larger volumes of the bladder and rectum were considered, the absolute difference in dose between the MVCT and kVCT plans increased. The same trend followed for hybrid plans compared to kVCT plans but to a lesser extent. Patient 2 showed the largest calculated differences in rectum and bladder dose for MVCT and hybrid plans compared to kVCT. Bladder dose difference of 47% and a rectal dose difference of 10% were seen in the MVCT plan for patient 2 at D_50_. The hybrid plans for patient 2 showed smaller differences of 35% and 7% at D_50_ for the bladder and rectum, respectively. Calculated doses to the PTV were similar in the three treatment plans calculated for each patient. For the three patients, the greatest difference in planned D_99_ (dose to 99% of the volume) in the PTV compared to kVCT plans was 2.6% and 0.5% for MVCT and hybrid plans, respectively.

**Table 1:** Calculated patient doses delivered to regions of interest for kVCT, MVCT and hybrid plans

		**Patient 1**			**Patient 2**			**Patient 3**		
	**Organ**	**kVCT**	**MVCT**	**Hybrid**	**kVCT**	**MVCT**	**Hybrid**	**kVCT**	**MVCT**	**Hybrid**
D_5MVCT_	B	67.1	63.7	67.6	81.8	77.2	80.9	78.6	80.3	78.7
	R	76.0	75.6	75.3	65.0	70.5	70.9	77.6	81.2	77.7
D_10MVCT_	B	62.5	60.8	62.3	75.1	75.6	75.3	77.0	77.1	77.1
	R	69.0	70.3	68.5	59.1	64.1	64.4	77.4	80.6	77.5
D_25MVCT_	B	48.1	48.4	48.4	50.7	57.1	55.6	67.8	67.8	68.1
	R	51.4	51.7	51.7	45.2	48.4	50.0	74.8	72.0	74.9
D_35MVCT_	B	41.2	39.9	42.3	39.4	49.0	46.0	58.8	59.5	59.0
	R	43.5	42.7	44.0	37.5	41.5	40.1	69.5	63.5	69.4
D_50MVCT_	B	30.9	33.0	31.9	25.4	37.3	34.2	48.1	47.2	48.1
	R	35.7	33.0	36.1	31.9	35.1	34.2	59.2	45.2	59.5
D_99_	P	72.2	72.8	72.2	74.2	73.7	74.6	71.9	73.8	71.8
D_1_	P	83.6	81.4	83.5	89.1	82.6	85.0	79.6	85.1	79.7

Doses to volumes of the planning target volume (PTV), bladder and rectum for treatment plans using original kVCT, MVCT, and hybrid image sets are shown. B = bladder, R = rectum, and P = PTV. D_nMVCT_ is the dose delivered to the absolute volume corresponding to n% of the ROI in the MVCT base plans.

The phantom “treatment” results are shown in Table 2. The kVCT plan with the O-MAR correction applied showed better agreement between the measured and calculated dose than the original image set, with a difference of -1.91% compared to -3.31%. However, both of these values were worse than the agreement with the hips removed, which was a difference of -1.0% for the kVCT plan. The measured doses for the MVCT plans were always lower than the calculated dose, by between -6.21% and -5.04% with and without the hip prostheses inserted. The best agreement was for the hybrid plans, which had differences ranging from -1.21% to -0.72% with the hips inserted and from -1.18% to -1.06% without them.

**Table 2:** Results of radiation delivery to the water tank phantom

**Image Set**	**Water**			**O-MAR kVCT**			**Original kVCT**		
	**Measured dose (Gy)**	**Planned dose (Gy)**	**% difference**	**Measured dose (Gy)**	**Planned dose (Gy)**	**% difference**	**Measured dose (Gy)**	**Planned dose (Gy)**	**% difference**
kVCT	2.01	2.03	-1.00	1.99	2.03	-1.91	1.97	2.04	-3.31
HybridCoarse	2.01	2.04	-1.14	2.02	2.03	-0.72	2.01	2.03	-1.21
HybridNormal	2.01	2.04	-1.06	2.00	2.02	-0.92	2.02	2.03	-0.75
HybridFine	2.01	2.03	-1.18	2.02	2.03	-0.74	2.01	2.03	-1.21
MV C	1.89	2.01	-6.21	1.91	2.01	-5.04			
MV N	1.91	2.03	-6.00	1.92	2.02	-5.41			
MV F	1.91	2.02	-5.73	1.91	2.03	-6.11			

Delivered dose was measured with a pinpoint ion chamber at a point corresponding to the center of the PTV and compared with the calculated dose at that point.

Table 3 contains the percent difference in dose to volumes of the bladder, rectum and PTV between the original plans and the recalculated verification dose for the kVCT and OMAR corrected kVCT image sets of the water tank phantom. The verification dose was higher than the original planned dose for every category of the OMAR image set and for the bladder and dose to 1% of the PTV in the uncorrected kVCT plan. The differences were larger for the kVCT plan without the OMAR correction, except for the D35 and D50 of the rectum.

**Table 3:** Percent difference between planned and verification dose for kVCT and OMAR corrected kVCT images

**Image set**	**kVCT**			**OMAR corrected kVCT**		
**Organ**	**Bladder**	**Rectum**	**PTV**	**Bladder**	**Rectum**	**PTV**
D5	1.23	-0.20		0.95	0.19	
D10	1.03	-0.57		0.87	0.12	
D25	0.86	-0.51		0.47	0.05	
D35	0.87	-0.06		0.61	0.33	
D50	0.76	-0.05		0.76	0.43	
D1			1.38			0.86
D99			-0.77			0.08

## Discussion

We have presented treatment planning results for plans calculated from patient data and measurements of treatment delivery using real hip prostheses within a water tank phantom designed to simulate patient anatomy. Our results explored the accuracy of using different sources of treatment planning images to treat patients with bilateral hip prostheses.

As follows from Table 1, there are differences in calculated dose between patient treatment plans depending on the source of treatment planning images. Contours and HT optimization settings were kept constant within patients, so the differences in calculated dose between plans were designed to be only the result of differences in the images used for treatment planning. Largest difference was observed between dose to D_50MVCT_ = 57 cm^3^ of the patient 2 bladder (Table 1). This patient had the strongest artifacts from his hip prostheses (Figure 1) resulting in a low effective density in this region. We found that the hybrid plans had the best agreement with the measured dose verified in phantom with or without the presence of the hip prostheses. In addition, recalculating kVCT phantom plans using the hybrid image set showed disparities on the order of the differences between the kVCT and hybrid plans. Due to the simplicity of the phantom geometry, the differences between plans were small; they could be larger for the more complicated patient anatomy.

Previous research has aimed at improving dose calculation accuracy in presence of high atomic number materials such as titanium hip prostheses using MVCT imaging. Hecox *et al.* investigated the accuracy of the HT dose calculation algorithm in the presence of a single hip prosthesis [9]. Similar to our results they found that there was very good agreement between calculated and delivered dose to the PTV. The accuracy of HT dose calculation is better in the presence of a single hip prosthesis. This is due to the increased freedom that the treatment planning software has to optimize the treatment plan. In the case of bilateral hip prostheses the software is very constrained because it tries to limit the number of beams that pass directly through the highly dense prostheses. The prostheses surround the target therefore there are only limited directions from which radiation can be effectively delivered. Our studies with the water tank phantom without the prostheses showed very good agreement between the measured and calculated dose for the plans based on the kVCT and the hybrid kVCT/MVCT image sets. This suggests that the phantom design is valid and that dose can be accurately calculated in water phantom.

A number of other factors may have also been responsible for the differences in measured dose observed during phantom irradiation. These include: artifacts present in the kVCT images, interpolation between slices in the hybrid image sets, use of different IVDT depending on the source of treatment planning images, the limited field of view of MVCT images, and uncertainty in the positioning of the ion chamber during measurement with respect to the image voxel where the calculated dose was reported. Metal artifacts in kVCT images alter image density values, especially in the target area for prostate cancer radiation therapy. Image density, quantified in HU is the basis for treatment planning and calculating the dose delivered to specific areas. The artifacts present in kVCT images created artificial regions of high and low density that did not accurately reflect true density values in the phantom. Our results show that the presence of metal artifacts did not have a significant effect on dose delivery to the PTV, rectum or bladder. It appears that metal artifacts are not the primary cause of treatment dose differences, perhaps due to the helical nature of HT radiation delivery.

As previously mentioned, hybrid kVCT/MVCT image sets were produced using the HT Planned Adaptive software. The 2, 4, and 6 mm slices of the MVCT studies were modified to 3 mm slices by interpolation to correspond with the slice spacing in the kVCT study. Decreased inter-slice spacing reduces the amount of interpolation necessary to create hybrid image sets and should result in image sets that more accurately reflect patient anatomy. However, the difference in the agreement between the measured and calculated dose for the coarse, normal and fine hybrid plans was minimal in our experiment, so the coarse setting would be the best to use, as it takes less time to perform the scan and delivers less radiation. Hybrid images have been shown to be suitable for HT dose re-computations with comparable accuracy to initial kVCT dose calculations [16]. This was reflected in our study as the DVH for kVCT and hybrid image plans were very similar and the calculated point doses in the PTV were nearly identical.

Depending on the source of images used to calculate treatment plans, different IVDT calibration curves were used to convert image HU values to electron density for treatment calculation. The two calibration curves are similar at low HU values from -1000 to 0 (equal to the densities of air and water, respectively) but differ significantly as greater HU values are considered. The hybrid image sets were comprised mostly of MVCT data; however there were portions of the images that were supplemented with kVCT data because the water tank phantom was larger than the 40 cm MVCT imaging diameter. Therefore, in the hybrid image treatment plans the electron density of the kVCT portions of the image may not have been accurately accounted for. In our study, the use of different IVDT calibration curves did not have a significant effect on dose delivery due to the relatively low densities of the materials used in the water tank phantom. The observed HU values of the water tank and water in the kVCT image were approximately zero so similar IVDT values were calculated compared to areas calculated with the MVCT calibration curve. The lateral edges of the tank were the only volume supplemented by the kVCT data, but there was much less fluence delivered to the PTV through these areas. This was because the smaller depth of the water relative to the width of the tank caused the optimizer to favour anterior and posterior gantry angles. However, the effects of using different calibration curves are expected to be more significant in patient treatment plans due to the greater density of muscle, bone and other tissue as well as greater variation in anatomy compared to the water tank phantom. If a large patient volume must be supplemented with kVCT data then there is the potential for large differences in IVDT which would have an effect on treatment delivery.

A limitation of using “MVCT only” for treatment plan calculations is the limited field of view of these images. MVCT allows for the clear visualization of pelvic anatomy in the presence of hip prostheses but large patients cannot be imaged entirely as in kVCT. In our study, the phantom was larger than the 40 cm imaging diameter of the HT MVCT unit therefore there were parts of the phantom that were not imaged. These non-imaged areas were not taken into account during treatment calculation. This may have contributed to plans based on MVCT images showing the largest dose differences in the PTV. Radiation was absorbed when delivered through the non-imaged areas, resulting in less dose reaching the target. This would explain why the plans based on MVCT images consistently had a difference between the measured and calculated dose of -5% to -6%, independently of whether or not the hips were inserted. Ruchala *et al.* have explored different algorithms to improve limited field of view images such as MVCT using planning CT data [17], similar to the method used by the Planned Adaptive Software to create hybrid image sets. The soft tissue contrast obtained in MVCT images is sufficient for contouring the prostate, bladder, and rectum; allowing for dose delivery verification with the Planned Adaptive software and plan adaptation if anatomic changes are clinically significant [18].

Investigation of different algorithms may prove helpful in improving the accuracy of hybrid images created from limited field of view MVCT images. Treatment plans should not be based on MVCT images alone for patients with significant volumes outside the 40 cm imaging region.

The factor that seems to have the largest impact on measured dose differences during phantom treatment is uncertainty in the positioning of the ion chamber. Registration of the phantom was performed prior to treatment in an effort to ensure accurate positioning of the ion chamber in specific voxels, however due to the relatively large size of the ion chamber relative to individual voxels there was potential for dislocation. This is significant due to the large dose differences that can exist between adjacent calculation voxels, especially in high dose gradient regions such as the bladder and rectum. For example, in the hybrid image plans the calculated dose immediately anterior and posterior of the expected point of measurement are on average approximately 6% higher and 6% lower respectively. This difference of 6% is roughly the observed experimental difference between calculated and measured dose. From this, it is difficult to determine if the true source of differences in measured dose, especially in the bladder and rectum due to dose inhomogeneity in these regions. The dose distribution calculated for the PTV is much more homogeneous and therefore it is expected that the ion chamber measurements in the PTV are much more accurate. The relative dose differences between adjacent voxels in the PTV are much smaller, typically < 1% and as such, the positioning of the ion chamber will not have a large effect on measured dose.

An emerging strategy for dealing with bilateral hip prostheses is the use of MR-only simulation [19,20]. Conceptually the work flow would be similar to incorporating MVCT through image fusion with the kVCT with similar concerns such as variable patient anatomy between scans and image fusion accuracy. Rosewall *et al.* found less inter-observer variability and no evidence of geometric distortion in an inter-observer study of prostate delineation on CT alone versus hybrid CT plus MRI in a cohort of patients with bilateral hip prosthesis but did not describe dosimetric results [21].

## Conclusion

In order to minimize the effect of the artifacts caused by high density metal hip implants in the prostate cancer patients, the following procedure is recommended: (i) a kVCT scan, followed by (ii) an MVCT scan using the coarse 6 mm slice spacing, and (iii) creation of a hybrid image set. MVCT image will provide better visualization of patient anatomy and hybrid kV/MVCT study enables more accurate calculations using updated MVCT relative electron density calibration. Ideally the kVCT and MVCT studies should be acquired in close proximity in order to minimize temporal variations in patient anatomy. If MVCT image fusion is not available, kVCT correction algorithms still provide improved dose calculation compared to uncorrected kVCT.

## Competing interests

The authors declare that they have no competing interests.

## Authors’ contributions

DC and SS carried out the measurements, participated in interpretation of data, and drafted the manuscript. RB, GB, and SY participated in study conception, interpretation of the data, and editing the manuscript. All authors read and approved the final manuscript.
